# Differential Activation of CD8^+^ Tumor-Specific Tc1 and Tc2 Cells by an IL-10-Producing Murine Plasmacytoma

**DOI:** 10.1155/1998/93545

**Published:** 1998

**Authors:** Christoph Specht, Hans-Gerd Pauels, Christian Becker, Eckehart Kölsch

**Affiliations:** Institute for Immunology University of Miinster Domagkstr. 3A Münster D-48129 Germany

**Keywords:** CD8^+ T cells, cytokine profiles, IFN-*γ*, plasmacytoma, T-cell subsets, tumor immunology

## Abstract

The involvement of counteractive CD8^+^ T-cell subsets during tumor-specific immune responses was analyzed in a syngeneic murine plasmacytoma model. CD8^+^ Tc cells against the immunogenic IL-10-producing BALB/c plasmacytoma ADJ-PC-5 can be easily induced by immunization of BALB/c mice with X-irradiated ADJ-PC-5 tumor cells *in vivo* and *in vitro*. However, the failure of recipient mice to mount a protective Tc response against the tumor during early stages of a real or simulated tumor growth is not due to immunological ignorance, but depends on the induction of tumor-specific tolerance, involving a population of tumorinduced CD8^+^ T cells that are able to inhibit the generation of tumor-specific Tc cells in a primary ADJ-PC-5-specific MLTC, using IFN-*γ* as a suppressive factor. Whereas most longterm cultivated CD8^+^ ADJ-PC-5-specific Tc lines produce type-1 cytokines on stimulation, at least two of them, which were derived from a primary MLTC, display a type-2 cytokine spectrum. Furthermore, the primary *in vitro* Tc response against ADJ-PC-5 cells shows characteristics of a Tc2 response. The Tc response is strictly depending on tumor-derived IL-10. CD8^+^ Tc cells that are induced in a primary MLTC do not produce IFN-*γ*, and the tumor-specific Tc response is enhanced by IL-4 but suppressed by IFN-*γ* or IL-12. In contrast, ADJ-PC- 5-specific CD8^+^ Tc cells from immunized mice are IFN-*γ* producing Tc1 cells. Since the primary *in vitro* Tc response against the tumor is suppressed even by the smallest numbers of irradiated ADJ-PC-5-specific Tc1 cells via IFN-*γ* these Tc1 cells behave similar to the suppressive CD8^+^ T cells that are induced during early stages of ADJ-PC-5 tumorigenesis.


*Developmental Immunology*, 1998, Vol. 6, pp. 331–342
Reprints available directly from the publisher
Photocopying permitted by license only

© 1998 OPA (Overseas Publishers Association)
N.V. Published by license under the
Harwood Academic Publishers imprint,
part of the Gordon and Breach Publishing Group
Printed in Malaysia

### Differential Activation of $CD8^+$ Tumor-Specific Tc1 and Tc2 Cells by an IL-10-Producing Murine Plasmacytoma

CHRISTOPH SPECHT*, HANS-GERD PAUELS, CHRISTIAN BECKER and ECKEHART KÖLSCH

*Institute for Immunology, University of Münster, Domagkstr. 3A, D-48129 Münster, Germany*

The involvement of counteractive $CD8^+$ T-cell subsets during tumor-specific immune responses was analyzed in a syngeneic murine plasmacytoma model. $CD8^+$ Tc cells against the immunogenic IL-10-producing BALB/c plasmacytoma ADJ-PC-5 can be easily induced by immunization of BALB/c mice with X-irradiated ADJ-PC-5 tumor cells *in vivo* and *in vitro*. However, the failure of recipient mice to mount a protective Tc response against the tumor during early stages of a real or simulated tumor growth is not due to immunological ignorance, but depends on the induction of tumor-specific tolerance, involving a population of tumor-induced $CD8^+$ T cells that are able to inhibit the generation of tumor-specific Tc cells in a primary ADJ-PC-5-specific MLTC, using IFN-$\gamma$ as a suppressive factor. Whereas most long-term cultivated $CD8^+$ ADJ-PC-5-specific Tc lines produce type-1 cytokines on stimulation, at least two of them, which were derived from a primary MLTC, display a type-2 cytokine spectrum. Furthermore, the primary *in vitro* Tc response against ADJ-PC-5 cells shows characteristics of a Tc2 response. The Tc response is strictly depending on tumor-derived IL-10. $CD8^+$ Tc cells that are induced in a primary MLTC do not produce IFN-$\gamma$, and the tumor-specific Tc response is enhanced by IL-4 but suppressed by IFN-$\gamma$ or IL-12. In contrast, ADJ-PC-5-specific $CD8^+$ Tc cells from immunized mice are IFN-$\gamma$ producing Tc1 cells. Since the primary *in vitro* Tc response against the tumor is suppressed even by the smallest numbers of irradiated ADJ-PC-5-specific Tc1 cells via IFN-$\gamma$, these Tc1 cells behave similar to the suppressive $CD8^+$ T cells that are induced during early stages of ADJ-PC-5 tumorigenesis.

*Keywords:* $CD8^+$ T cells, cytokine profiles, IFN-$\gamma$, plasmacytoma, T-cell subsets, tumor immunology

### INTRODUCTION

In most cases, tumors grow in their primary hosts or syngeneic recipients without beeing hampered by the immune system, even if the tumor cells express immunogenic neoantigens. The lack of apparent immunogenicity of tumors *in situ* might be due to special properties of the tumor cells, for example lack of costimulatory molecules, downregulation of MHC molecules, or production of immunosuppressive

*Corresponding author. Tel.: +49-251-83-56286, Fax: +49-251-83-56285, E-mail: specht@uni-muenster.de.

factors (Browning and Bodmer, 1992; Chen et al., 1992; Sulizeanu, 1993), or due to intrinsic tolerance mechanisms of the immune system (Mengersen et al., 1975; Naor, 1979; North, 1985). Under special experimental conditions, however, it is possible to mount protective immune responses against tumor cells, involving specific Tc cells or nonspecific macrophages and NK cells. Activation of Tc cells against tumor-specific transplantation antigens is necessary for any kind of vaccinization strategy or for adoptive immunotherapy against cancer. Thus, in the context of such therapies, it is necessary to analyze phenotypes and activation modalities of tumor-specific Tc cells and their fate *in situ*, in the presence of a growing tumor. Using the highly malignant BALB/c plasmacytoma ADJ-PC-5 as a model tumor, we studied the regulation of tumor-specific Tc responses, especially during early stages of tumorigenesis. Syngeneic BALB/c mice can be protectively immunized against this tumor by four weekly i.p. injections with $10^7$ X-irradiated ADJ-PC-5 cells (Cihak et al., 1981), which leads to the activation of tumor-specific $CD8^+$ Tc cells. Specific $CD8^+$ Tc cells against the tumor can also be induced *in vitro*, in a primary syngeneic mixed lymphocyte tumor cell culture (MLTC) (Haubeck and Kölsch, 1982). However, the growth of ADJ-PC-5 tumor cells in syngeneic BALB/c mice is accompanied by the induction of a subset of $CD8^+$ regulatory T cells, which are characterized by their ability to suppress the induction of tumor-specific $CD8^+$ Tc cells in a primary MLTC *in vitro* (Haubeck and Kölsch, 1982, 1986). These suppressive T cells are induced during early stages of tumorigenesis, when the tumor burden is small, and possibly hold down a protective cytotoxic T-cell (Tc) response against the tumor, even when the tumor mass reaches an immunogenic level. Recent data indicate that the T-cell-mediated suppressive effect *in vitro* involves IFN-$\gamma$ as a suppressor factor, but does not depend on IL-2 consumption (Pauels et al., 1996). The finding that IFN-$\gamma$ is a suppressive factor for Tc induction *in vitro* prompted us to study phenotypes and activation modalities of ADJ-PC-5-specific Tc cells in more detail.

### RESULTS

### Simulation of ADJ-PC-5 Tumor Growth in the Peritoneal Cavity is Accompanied by the Induction of IFN-$\gamma$-Producing Suppressive $CD8^+$ T Cells

In previous studies, an *in vitro* protocol for the induction of ADJ-PC-5-specific Tc cells was developed, which is based on a primary syngeneic MLTC (Haubeck and Kölsch, 1982). As shown in Figure 1(a), tumor-induced suppressive T cells can be isolated from the peritoneal cavity of syngeneic BALB/c mice that have been subjected to a simulated ADJ-PC-5 tumor growth by daily i.p. injections of exponentially increasing numbers of 60-Gy X-irradiated ADJ-PC-5 cells. These T cells are characterized by their ability to suppress the induction of a tumor-specific Tc response from naive BALB/c spleen cells in a primary syngeneic ADJ-PC-5-specific MLTC *in vitro*. The respective suppressive T cells have been shown previously to be $CD8^+$ and to display no substantial cytolytic activity against ADJ-PC-5 cells (Haubeck and Kölsch, 1982, 1986). Such suppressive cells are not found in the peritoneal cavity of naive mice. The dominant role played by IFN-$\gamma$ as a suppressive factor in this experimental system is proven by two lines of evidence: (1) Addition of recombinant IFN-$\gamma$ to a primary ADJ-PC-5-specific MLTC substantially reduces Tc induction against the tumor cells; see Figure 1(c). (2) Addition of IFN-$\gamma$-specific monoclonal antibodies to an MLTC containing PEC from tumor-treated mice can almost completely restore Tc induction, whereas addition of control monoclonal antibodies has no such effect; see Figure 1(d).

### Characterization of the ADJ-PC-5-Specific Tc Response in a Primary MLTC

Since the primary *in vitro* Tc response against ADJ-PC-5 plasmacytoma cells is suppressed by tumor-induced $CD8^+$ regulatory T cells via IFN-$\gamma$, the effects of other exogenous and endogenous cytokines on a primary MLTC as well as the phenotype of the resulting Tc cells were analyzed in more detail.

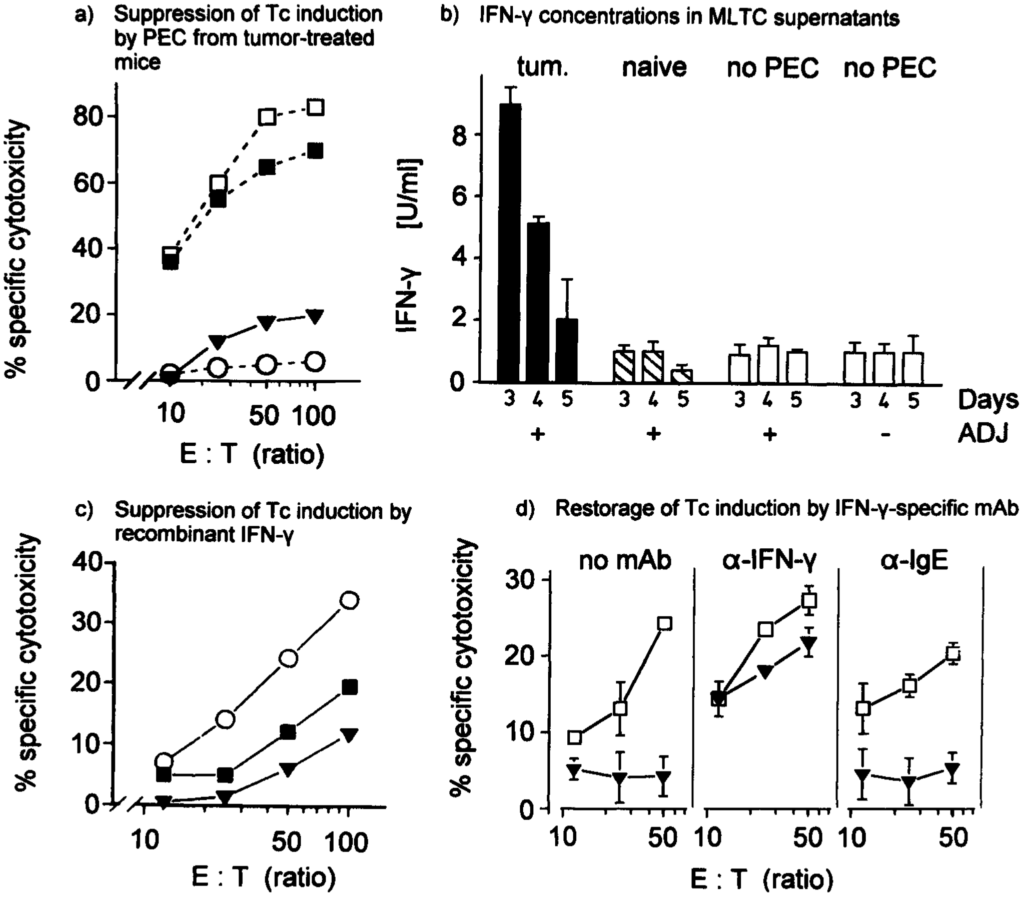

FIGURE 1 Suppression by PEC from ADJ-PC-5-treated mice is due to IFN-γ. (a) Suppressive T cells were generated by repeated i.p. injections of exponentially increasing numbers of X-irradiated ADJ-PC-5 cells (according to Materials and Methods) and added to an ADJ-PC-5-specific MLTC on day 0. The resulting tumor-specific cytotoxicity was measured on day 6 by a $^{51}$Cr-release assay. The cytotoxicity in the presence of nonadherent PEC from tumor treated mice (▼—▼) was compared with the cytotoxicity in the presence of PEC from naive mice (■—■). Control cultures were set up without PEC (□—□) or without PEC and ADJ-PC-5 cells (O—O). (b) Cytokine kinetics in MLTC supernatants corresponding to part (a). Substantial amounts of IFN-γ can only be detected in MLTC supernatants containing suppressive T cells from tumor-treated mice (tum.). Samples of culture supernatants were taken at the indicated time points and assaged for IFN-γ according to Materials and Methods. (c) MLTC cultures were set up with $2 \times 10^7$ navie BALB/c spleen cells and $1 \times 10^6$ X-irradiated ADJ-PC-5 cells in the presence of different concentrations IFN-γ: O: no IFN-γ added; ■: 50 U/ml; and ▼ 500 U/ml. Specific cytotoxicity was measured on day 6 of the culture period. (d) MLTC cultures were set up with (▼—▼) or without (□—□) nonadherent PEC from tumor-treated mice in the presence of 20 μg/ml of an IFN-γ-specific monoclonal rat IgG antibody (α-IFN-γ) or in the presence of 20 μg/ml of an irrelevant IgE-specific rat IgG monoclonal antibody (α-IgE).

Beside the suppressive effect of IFN-γ on Tc induction *in vitro*, addition of IL-4 to a primary ADJ-PC-5-specific MLTC has an enhancing effect on Tc induction, whereas addition of IL-12 is slightly suppressive in this context (Becker et al., 1997). Another striking difference between the primary *in vitro* Tc response against ADJ-PC-5 cells and a typical Tc response, as seen in the case of a primary allogenic MLC, is the absolute dependence of the tumor-specific Tc response on tumor-derived IL-10. As shown in Figure 2, addition of IL-10-specific monoclonal antibodies to a primary MLTC can completely eliminate the ADJ-PC-5-specific Tc response, whereas an allogenic control MLC of CBA/J spleen cells against irradiated BALB/c spleen cells is not affected. In addition, cytokine production of $CD8^+$

T cells from a primary ADJ-PC-5-specific MLTC and a primary allogenic MLC was compared on a single-cell level by flow cytometry. As shown in Figure 3, the percentage of CD8$^+$ T cells increases significantly in both types of cultures as compared with unstimulated control cultures, indicating that an antigen-specific stimulation of CD8$^+$ T cells must have taken place in both types of cultures. Despite the development of a cytotoxic phenotype, CD8$^+$ T cells from an ADJ-PC-5-specific MLTC differ from typical alloantigen-induced CD8$^+$ Tc cells by a lack of IFN-$\gamma$ production. Production of IL-4 could neither be

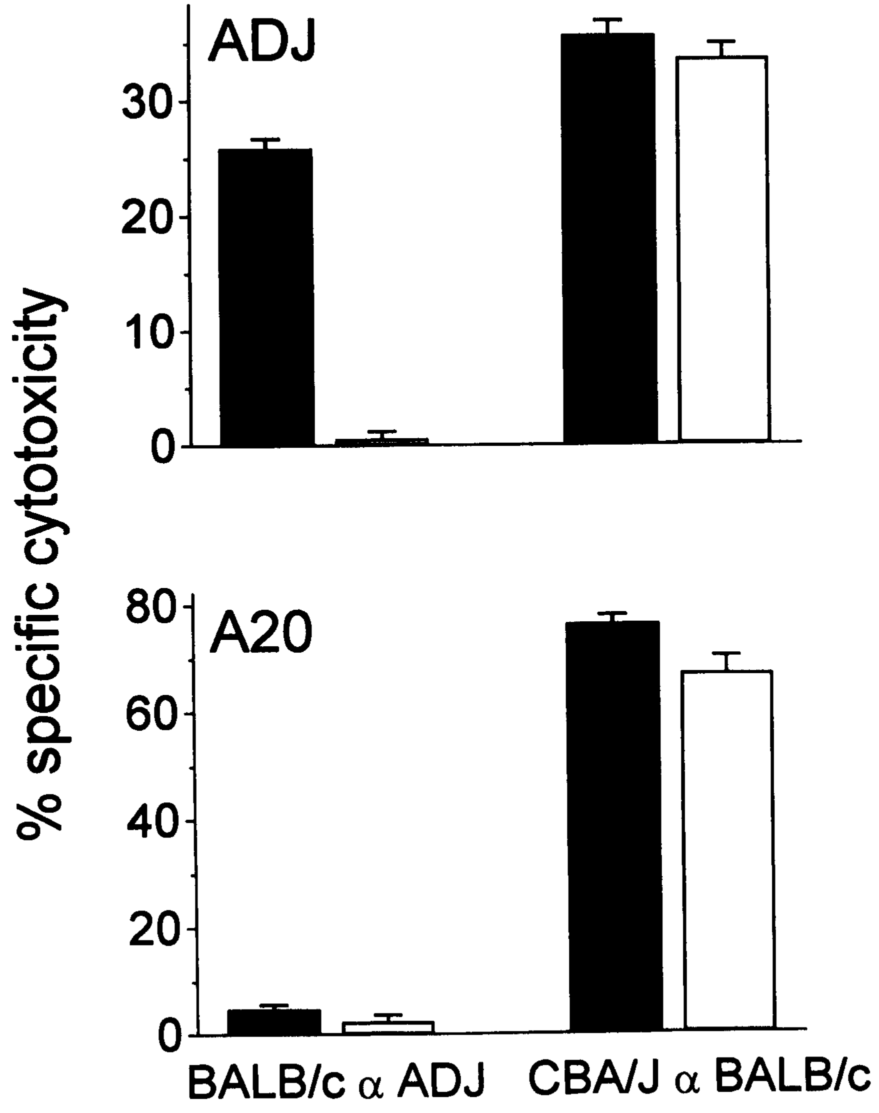

FIGURE 2 Induction of ADJ-PC-5-specific Tc cells in a primary MLTC depends on tumor-derived IL-10. MLTC cultures (BALB/c $\alpha$ ADJ) were set up with $2 \times 10^7$ BALB/c spleen cells and $1 \times 10^6$ X-irradiated ADJ-PC-5 cells in the absence (closed bars) or presence (open bars) of a mix of two IL-10-specific monoclonal antibodies (SXC-1 and SXC-2; 10 $\mu$g/ml each). Allogenic MLC cultures (CBA/J $\alpha$ BALB/c) were set up in the presence or absence of IL-10-specific antibodies with $2 \times 10^7$ CBA/J spleen cells and $1 \times 10^7$ X-irradiated BALB/c spleen cells. The resulting cytotoxicity was measured on day 6 against ADJ-PC-5 (ADJ) cells and a syngeneic control B-cell tumor (A20).

detected in MLTC cultures nor in MLC cultures. However, it must be mentioned that at least some CD8+ T-cell lines that were derived from a primary MLTC by repeated *in vitro* stimulation with irradiated ADJ-PC-5 cells produce substantial amounts of IL-4 but no IFN-$\gamma$ and aquire a full Tc2 phenotype (see what follows).

All three features of the primary tumor-specific Tc response, that is, the suppression by IFN-$\gamma$ and IL-12, the dependence on IL-10, and the lack of IFN-$\gamma$ production by the resulting tumor-specific Tc cells, point toward a type-2 nature of the *in vitro* Tc response against the tumor.

### Characterization of Long-Term Cultivated ADJ-PC-5-Specific CD8+ Tc Lines

In order to phenotypically characterize ADJ-PC-5-specific Tc cells further, CD8+ Tc lines were generated from spleen cells of ADJ-PC-5-immunized BALB/c mice or from spleen cells of naive BALB/c mice that were stimulated with irradiated ADJ-PC-5 cells in a primary syngeneic MLTC. Nine of initially 24 CD8+ ADJ-PC-5-specific Tc lines could be cultivated over a period of more than 3 months; among them, seven developed a quite homogeneous phenotype. As shown in Table I, all of the nine lines specifically lyse ADJ-PC-5 cells but not cells of

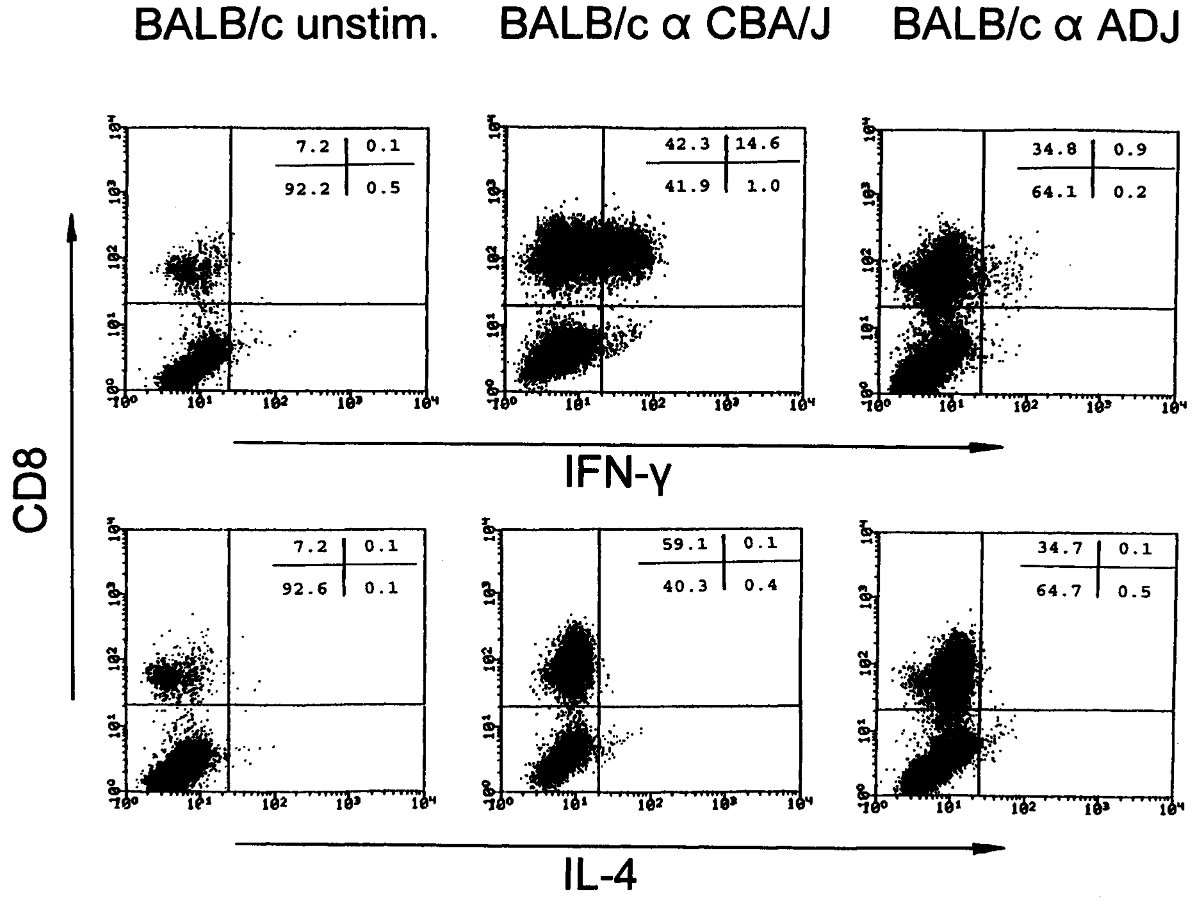

FIGURE 3 Flow cytometric detection of intracellular cytokines on a single-cell level. Living cells were isolated from MLTC or allogeneic MLC cultures after day 5 of the culture period and stained for intracellular cytokines according to Materials and Methods. Both cytokine-specific MoAb are of the same isotype and were used at the same concentrations. Thus, data for an isotype control antibody are not shown. Percentages of the respective populations are indicated in the upper right quadrant of each plot.

TABLE I Phenotypes of ADJ-PC-5-Specific Tc Lines

| Line | Preimmunization[a] | % specific cytotoxicity[b] | | | % CD8[+c] | Vβ usage[d] | | Cytokine production[e] | | | |
|---|---|---|---|---|---|---|---|---|---|---|---|
| | | Target | E:T | | | | | IFN-γ | | IL-4 | |
| | | | 10:1 | 5:1 | | major Vβ | % Vβ/CD8+ | — | αCD3 | — | αCD3 |
| BTc 2 | $10^7$ (7×) | ADJ | 78 | 63 | 97 | 6 | 98 | 0 | 8,439 | 0 | 998 |
| | | Meth. A | 13 | 5 | | | | | | | |
| BTc 3 | - | ADJ | 56 | 33 | 88 | 6 | 99 | 0 | 1,717 | 0 | 22,579 |
| | | Meth. A | 0 | 0 | | | | | | | |
| BTc 4 | 5 × $10^6$ (1×) | ADJ | 42 | 20 | 90 | 6 | 94 | 0 | 31,129 | 0 | 1,030 |
| | | Meth. A | 0 | 0 | | | | | | | |
| BTc 5 | 5 × $10^6$ (1×) | ADJ | 58 | 47 | 68 | 6 | 90 | 0 | 13,311 | 0 | 692 |
| | | Meth. A | 18 | 11 | | | | | | | |
| BTc 6 | 5 × $10^6$ (1×) | ADJ | 72 | 56 | 95 | 6 | 34 | 0 | 27,760 | 0 | 977 |
| | | Meth. A | 13 | 10 | | | | | | | |
| BTc 7 | - | ADJ | 41 | 30 | 40 | 8.1/8.2 | 63 | 0 | 974 | 0 | 23,893 |
| | | Meth. A | 5 | 3 | | | | | | | |
| BTc 8 | - | ADJ | 55 | 28 | 95 | 8.1/8.2 | 76 | 0 | 3,500 | 0 | 0 |
| | | Meth. A | 1 | 0 | | | | | | | |
| | 0 | BTc 9 | - | ADJ | 57 | 32 | 94 | 8.1/8.2 | 78 | 0 | 3,1200 |
| | | Meth. A | 0 | 0 | | | | | | | |
| BTc 14 | - | ADJ | 60 | 27 | 95 | 8.1/8.2 | 62 | 0 | 3,300 | 0 | 0 |
| | | Meth. A | 0 | 0 | | | | | | | |

[a]Tc lines were generated from spleen cells of naive or ADJ-PC-5 preimmunized BALB/c mice.[b]Cytotoxicity of Tc lines was measured against $10^4$ ADJ-PC-5 or Meth A cells in a 6-hr $^{51}$Cr-release assay at the indicated effector: target ratios.[c]Total percentage of CD8+ within the respective line.[d]Dominant Vβ TcR type and percentage of T cells espressing this Vβ type among CD8+ T cells.[e]T cells were cultivated at a density of 1 × $10^6$/ml for 2 days and stimulated with either immobilized αCD3 antibodies or left unstimulated.

syngeneic control tumors like the BALB/c fibrosarcoma Meth A, which expresses MHC class I molecules at about the same density as ADJ-PC-5 cells. T cells of all lines were examined for the expression of TcR Vβ elements 2, 3, 5.1, 5.2, 6, 7, 8.1, 8.2, 8.3, 9, 11, and 17a.

Four of these lines (BTc2, BTc3, BTc4, and BTc5) consist almost entirely of Vβ6+CD8+ cells, whereas the majority of CD8+ cells in lines BTc7, BTc8, BTc9, and BTc14 are of the Vβ8.1/8.2 phenotype. CD8+ cells of line BTc6 are 34% Vβ6+; the remaining CD8+ cells express lower percentages of other TcR Vβ families. Early analysis of TcR expressed by the unstable lines revealed that even these lines contained CD8+ T cells of the Vβ6+ or Vβ8.1/8.2+ type at frequencies significantly higher than among naive peripheral T cells (data not shown).

Lines BTc3 and BTc7, which were derived from a primary MLTC, produce a type-2 cytokine spectrum (IL-4) on stimulation, whereas the other lines produce type-1 cytokines, including line BTc2, which was derived from a hyperimmune BALB/c mouse, and lines BTc4, BTc5, and BTc6, which were derived from once preimmunized mice. The fact that at least two out of five Tc lines derived from primary MLTC cultures by repeated *in vitro* restimulation develop a Tc2 phenotype in the absence of exogenous IL-4 or IFN-γ-specific monoclonal antibodies, is in concordance with a type-2 nature of the primary *in vitro* Tc response against the tumor.

### Phenotypic Analysis of CD8+ T Cells from ADJ-PC-5 Immune BALB/c Mice

As demonstrated before, all ADJ-PC-5-specific Tc lines derived from preimmunized BALB/c mice are Tc1 cells, whereas some of the Tc lines derived from primary MLTC cultures are Tc2 cells. Since the phenotype, and especially the cytokine profile of cultivated T cells can be strongly influenced by the culture conditions, it had to be tested if a comparable Tc1/Tc2 pattern could also be found among peripheral T cells from ADJ-PC-5 immune BALB/c mice. Table II shows the results from a three-color fluores-

TABLE II Flow Cytometric Analysis of *in vivo* Induced ADJ-PC-5-Specific Tc Cells

| Tc induction | % $CD8^+$ | % $V\beta6^+/CD8^+$ | % IFN-$\gamma^+/CD8^+$ | % IFN-$\gamma^+/CD8^+$ $V\beta6^+$ | % IL-$4^+/CD8^+$ |
|---|---|---|---|---|---|
| *In Vivo* | | | | | |
| Spleen naive | 13.5 | 9.5 | 0.3 | 0.3 | 0.06 |
| Spleen immune | 14.9 | 9.3 | 3.8 | 3.1 | 0.08 |
| PEC naive | 8.0 | 10.1 | 0.8 | 0.9 | 1,24 |
| PEC immune | 9.8 | 12.0 | 7.1 | 12.0 | 1.67 |

Spleen cells or PEC were pooled from three individual ADJ-PC-5 immune or naive BALB/c mice and analyzed by flow cytometry according to Materials and Methods.

cence analysis of spleen cells and PEC from naive and ADJ-PC-5 immune BALB/c mice stained for CD8, $V\beta6$, IFN-$\gamma$, and IL-4. Repeated immunization of BALB/c mice with the tumor does not substantially alter the total number of splenic $CD8^+$ or $CD8^+V\beta6^+$ T cells, which is a main phenotype of ADJ-PC-5-specific Tc lines, but induces IFN-$\gamma$ production in a small but significant fraction of $CD8^+$ and $CD8^+V\beta6^+$ T cells. The data demonstrate that the actual percentage of ADJ-PC-5-specific Tc cells among $CD8^+$ or $CD8^+V\beta6^+$ T cells must be low, even in immune mice. However, production of IFN-$\gamma$ by the respective cells identifies them as Tc1 cells. Production of IL-4 is only detectable in a small fraction of $CD8^-$ spleen cells from immune mice, but not in $CD8^+$ cells. Peritoneal T cells from immune and naive BALB/c mice display similar properties, as seen for splenic T cells. As for spleen cells, immunization with the tumor increases the percentage of IFN-$\gamma$-producing $CD8^+$ or $CD8^+V\beta6^+$ T cells by a factor of about 10. A relatively high percentage of peritoneal $CD8^+$ T cells produces IL-4. However, this population of IL-4-producing peritoneal $CD8^+$ T cells does not expand during immunization, indicating that the tumor-specific Tc response in the peritoneal cavity is a Tc1 response, too.

### ADJ-PC-5-Specific Tc1 Cells Are Potent Suppressors of Tc Induction in a Primary ADJ-PC-5-Specific MLTC *In Vitro*

Since IFN-$\gamma$ is a suppressive factor for Tc induction in a primary ADJ-PC-5-specific MLTC, we analyzed whether this suppressive effect is also exerted by ADJ-PC-5-specific Tc1 cells. As shown in Table III, a substantial amount of Tc suppression in a primary MLTC is seen in the presence of even the lowest numbers of X-irradiated ADJ-PC-5-specific Tc1 cells. The suppressive effect in this case must be due to IFN-$\gamma$, since addition of IFN-$\gamma$-specific monoclonal antibodies to the cultures can in part avoid suppression (Table III, Exp. III). Irradiation of the Tc1 cells is necessary, because otherwise the cultures would be overgrown by restimulated Tc1 cells within a few days, making an analysis of the primary *in vitro* Tc response impossible. Suppression of the primary Tc response by a rapid elimination of ADJ-PC-5 stimulator cells can be excluded in the case of low numbers of irradiated Tc1 cells, since at any time point almost equal numbers of ADJ-PC-5 stimulator cells could be visually detected in MLTC cultures, irrespective if irradiated Tc1 cells were added or not. In contrast to low numbers of irradiated Tc1 cells, suppression by high numbers of irradiated Tc1 cells is not influenced by IFN-$\gamma$-specific mAb (data not shown). Suppression of the primary *in vitro* Tc response in this case, or in the presence of high numbers of irradiated Tc2 cells, can be entirely related to a rapid elimination of ADJ-PC-5 stimulator cells within the first hours of the culture period. The data demonstrate that Tc1 cells can interfere with the generation of ADJ-PC-5-specific Tc cells in a primary syngeneic MLTC using IFN-$\gamma$ as a suppressor factor. Thus, these ADJ-PC-5-specific Tc1 cells behave similarly to tumor-induced suppressive T cells from ADJ-PC-5 tumor-bearing mice (Pauels et al., 1996).

### DISCUSSION

Although the BALB/c plasmacytoma ADJ-PC-5 is highly immunogenic, tumor growth in syngeneic

TABLE III Suppression of ADJ-PC-5-Specific Tc Induction in a Primary MLTC by ADJ-PC-5-Specific Type-1 Tc Lines

| | Stimulator | Anti-IFN-$\gamma$ | Type-1 Tc | % specific cytotoxicity against ADJ-PC-5 100:1 | 50:1 | 25:1 |
|---|---|---|---|---|---|---|
| Exp. I | — | — | — | 0 | 0 | 0 |
| | ADJ | — | — | 46 | 40 | 15 |
| | ADJ | — | BTc 2 ($1 \times 10^5$) | 0 | 0 | 0 |
| | ADJ | — | BTc 2 ($5 \times 10^5$) | 0 | 0 | 0 |
| Exp. II | — | — | — | 0 | 0 | 0 |
| | ADJ | — | — | 29 | 19 | 10 |
| | ADJ | — | BTc 4 ($1 \times 10^5$) | 0 | 0 | 0 |
| | ADJ | — | BTc 4 ($5 \times 10^5$) | 0 | 0 | 0 |
| Exp. III | ADJ | — | — | 70 | 61 | 54 |
| | ADJ | 20 $\mu$g/ml | — | 70 | 64 | 40 |
| | ADJ | — | BTc 8 ($1 \times 10^5$) | 8 | 10 | 1 |
| | ADJ | 20 $\mu$g/ml | BTc 8 ($1 \times 10^5$) | 36 | 26 | 16 |
| | ADJ | 5 $\mu$g/ml | BTc 8 ($1 \times 10^5$) | 4 | 9 | −8 |

Thirty-Gy X-irradiated type-1 Tc cells were added on day 0 at the indicated cell numbers to a primary ADJ-PC-5-specific MLTC containing different concentrations of IFN-$\gamma$-specific monoclonal antibodies. The resulting ADJ-PC-5-specific cytotoxicity was measured on day 6 in a $^{51}$Cr-release assay at the indicated effector:target ratios.

BALB/c mice does not elicit a protective tumor-specific Tc response, but induces a state of tumor-specific tolerance. We could previously demonstrate that the tumor induces a population of CD8$^+$ T cells during early stages of tumorigenesis, which are able to suppress a primary ADJ-PC-5-specific Tc response *in vitro* through IFN-$\gamma$ (Pauels et al., 1996).

Recent data concerning Tc cells imply a functional and phenotypical dichotomy among CD8$^+$ T cells (LeGros and Erard, 1994; Sad et al., 1995), similar to the one observed in CD4$^+$ Th1 and Th2 cells. Since suppressive T cells from tumor-tolerant mice and Tc cells from tumor-immune mice are both CD8$^+$, we asked whether the failure of BALB/c mice to mount a protective Tc response against a growing ADJ-PC-5 tumor might reflect the involvement of counteractive CD8$^+$ Tc1 and Tc2 cells. To answer this question, the phenotypes and cytokine profiles of ADJ-PC-5-specific T cells had to be analyzed.

Long-term cultivated ADJ-PC-5-specific Tc cells are CD8$^+$ and use TcR of the V$\beta$6 and V$\beta$8 types. The exclusive CD8$^+$ phenotype of the lines is in accordance with the finding that ADJ-PC-5 cells are MHC class II negative (Becker et al., 1997). The majority of the Tc lines produce high amounts of IFN-$\gamma$ but no or only marginal amounts of IL-4 on stimulation, and can thus be classified as Tc1 cells (Sad et al., 1995). Nevertheless, at least two of five ADJ-PC-5-specific CD8$^+$ T-cell lines, which were derived from a primary MLTC by repeated restimulation with X-irradiated tumor cells, are IL-4-producing Tc2 cells. Whereas in other experimental systems, the addition of high amounts of IL-4 and blocking antibodies against IFN-$\gamma$ is a prerequisite for the generation of antigen-specific Tc2 lines (Sad et al., 1995), the exogenous addition of immunomodulating cytokines or antibodies against cytokines is not necessarily required for the induction of tumor-specific Tc2 cells against ADJ-PC-5 plasmacytoma cells. The driving force for Tc2 differentiation in this case seems to be endogenous IL-10 produced by the tumor cells themselves, since addition of blocking antibodies against IL-10 to a primary MLTC can completely abrogate the induction of tumor-specific Tc cells (see what follows). Since the cytokine phenotype of long-term cultivated T-cell lines is strongly influenced by the culture conditions, the observed numbers of Tc1 and Tc2 lines must not necessarily reflect the participation of both T-cell subsets during tumor-specific Tc responses *in situ*. For this reason, we examined the involvement of either of these T-cell subsets among CD8$^+$ and CD8$^+$V$\beta$6$^+$ T cells during ADJ-PC-

5-specific Tc responses *in vivo* and *in vitro* using intracellular cytokine staining ($CD8^{+}V\beta6^{+}$ is a major phenotype of ADJ-PC-5-specific Tc lines).

ADJ-PC-5-specific Tc cells from immunized mice are Tc1 cells. This is proven by the exclusive generation of ADJ-PC-5-specific Tc1 lines from preimmunized mice. Furthermore, immunization with the tumor significantly increases the percentages of IFN-$\gamma$-producing $CD8^{+}$ and $CD8^{+}V\beta6^{+}$ T cells among spleen cells and PEC, as compared with naive mice.

In contrast to *in vivo* induced ADJ-PC-5-specific Tc cells, the *in vitro* Tc response against the tumor shows Tc2 characteristics. This is demonstrated by a lack of IFN-$\gamma$ production of the tumor-reactive Tc cells, which are induced during a primary MLTC. It is also confirmed by means of supplementation experiments with exogenous cytokines, since the primary *in vitro* Tc response against ADJ-PC-5 cells is enhanced by IL-4, but suppressed by IFN-$\gamma$ and IL-12. Furthermore, the primary *in vitro* Tc response against ADJ-PC-5 is suppressed by irradiated Tc1 cells via IFN-$\gamma$.

In this context, the Tc1 cells behave similar to the previously described $CD8^{+}$ suppressive T cells from tumor-tolerant mice (Pauels et al., 1996). However, these suppressive $CD8^{+}$ T cells did never show any significant cytotoxicity against ADJ-PC-5 cells, whereas the Tc1 lines are potent tumor-specific Tc cells. It is presently not clear if the suppressive T cells in tumor-tolerant mice are Tc1 cells that *in situ* occur at frequencies too low to cause any substantial cytotoxicity or if they are incompletely activated Tc1 cells, which produce suppressive cytokines but are not sufficiently activated for cytotoxicity.

IL-10 has been described as a cytotoxic T-cell differentiation factor (Chang and Zlotnik, 1991), as well as an inhibitor of $CD8^{+}$ cytotoxic T-cell responses in primary allogenic MLC cultures (Bejarano et al., 1992). Thus, the effects of IL-10 on $CD8^{+}$ T cells seem to depend on the kind of $CD8^{+}$ T-cell response studied. In our experimental system, tumor-derived IL-10 is an essential differentiation factor for the induction of $CD8^{+}$ ADJ-PC-5-specific Tc cells, since blocking of IL-10 in MLTC cultures by monoclonal antibodies can completely abrogate the tumor-specific Tc response.

Production of IL-10 is a general property of malignancies of the B-lymphocyte lineage (Bost et al., 1995). It has previously been shown for $CD4^{+}$ T cells that IL-10, which is produced by Th2 cells, is a potent inhibitor of Th1 functions (Fiorentino et al., 1989), whereas Th1 cells are able to suppress Th2 cells via IFN-$\gamma$ (Gajewski and Fitch, 1988). It is also known that Th1 functions are drastically decreased in mice bearing IL-10-producing plasma-cell tumors (Ruzek and Mathur, 1995). Therefore, it is likely that production of IL-10 by ADJ-PC-5 cells strongly influences the $CD8^{+}$ Tc-cell response against the tumor. In this context, high cell numbers of ADJ-PC-5 cells, which are present in a MLTC produce sufficiently high amounts of IL-10 to dictate a Tc2-dominated response against the tumor *in vitro*. On the other hand, low numbers of tumor cells, which are present during early stages of tumorigenesis *in vivo*, could result in a Tc1-dominated response due to a lack of substantial amounts of IL-10. The same seems to be true for mice that were weekly immunized with high numbers or X-irradiated ADJ-PC-5 tumor cells. In this case, the IL-10 might be rapidly cleared from the surrounding of the tumor cells without being further produced by the tumor cells that die from irradiation.

Suppression via IFN-$\gamma$ of the IL-10-driven Tc2 response against ADJ-PC-5 cells is the main reason for the downregulation of the tumor-specific Tc response in a primary MLTC by $CD8^{+}$ T cells from tumor-treated mice. However, the question remains to be answered, how an early response of IFN-$\gamma$-producing $CD8^{+}$ T cells during early stages of tumorigeneses can omit the onset of a Tc1-dominated response during the course of a subsequent immunization with high numbers of tumor cells.

We are presently favoring the following model: During early stages of tumorigenesis, when no substantial amounts of IL-10 are present, the growing tumor induces a transient tumor-specific Tc1 response. This early Tc1 response, which is not strong enough to cause a regression of the tumor, is subsequently stopped by tumor-derived IL-10, when the tumor mass increases. These Tc1 cells must be in some state of anergy, because a subsequent immunization with high numbers of X-irradiated ADJ-PC-5 tumor cells can no longer induce a protective tumor-specific Tc1 response, as it would be the case in naive mice. The Tc1 cells are likely to be anergized, but not deleted by the tumor, since $CD8^+$ T cells from tumor-bearing mice, or from mice that have been subjected to a simulated tumor growth, can still suppress a Tc2-dominated primary MLTC via IFN-$\gamma$.

### MATERIALS AND METHODS

### Mice and Tumors

Female BALB/c mice were aged 8 to 12 weeks at the beginning of each experiment. The animals were either purchased from Charles River (Sulzfeld, Germany) or bred at our own animal facility. The nonsecreting variant of the BALB/c plasmacytoma ADJ-PC-5 (ADJ-PC-5-NS) (Blatt and Haïmovich, 1977), originally obtained from J. Haïmovich (Weizmann Institute, Rehovot, Israel) was used as a model tumor. The BALB/c fibrosarcoma Meth A (Old et al., 1962), the DBA/2 plasmacytoma ULMC (Bosslet et al., 1979), and the BALB/c B-cell lymphoma A20 (Kim et al., 1979) were used as control tumors in some experiments.

### Media

Dulbecco's modified Eagle's medium, containing penicillin (50 U/ml), streptomycin (50 $\mu$g/ml), L-glutamine (2 mM), 2-mercaptoethanol ($5 \times 10^5$ M), nonessential amino acids, 10% FCS, and additionally 3.5 g/l glucose (referred to as MLC medium), was used for most experiments. Hank's balanced salt solution (BSS) was used as washing medium.

### Monoclonal Antibodies

Monoclonal rat antibodies specific for murine IFN-$\gamma$ (R4-6A2, IgGl; (Spitalny and Havell, 1984), V$\beta$6 (44-22-1, IgG2a; (Acha-Orbea et al., 1985), and V$\beta$8.1 (KJ 16, IgG2a; Haskins et al., 1984) were used as hybridoma supernatant or as affinity-purified material using Protein G sepharose (Pharmacia, Uppsala, Sweden). Rat monoclonal antibodies against IL-10 (SXC-1 and SXC-2, IgM; Mosmann et al., 1990) were purified from hybridoma supernatants by ammonium sulphate precipitation followed by ion-exchange chromatoraphy on hydroxylapatite columns.

### Generation of Tumor-Induced Suppressive T Cells by Tumor Growth Simulation

The *in vivo* induction of suppressive T cells by tumor growth simulation has been previously described (Haubeck and Kölsch, 1982). Briefly, BALB/c mice recieved daily intraperitoneal injections of exponentially increasing numbers of 60-Gy X-irradiated ADJ-PC-5 cells starting with eight cells on day 0. According to the generation time of the tumor, doses of injected tumor cells were doubled every day, until mice had recieved $10^5$ cells on day 16. Eight days after the last treatment with ADJ-PC-5 cells, peritoneal exudate cells (PEC) were collected by a peritoneal lavage and fractionated into adherent and nonadherent cells by overnight incubation on plastic dishes at 37°C in MLC medium. Mice in which tumor growth was simulated by the preceding protocol are subsequently denoted "tumor-treated."

### Generation and Culture of ADJ-PC-5-Specific Tc Lines

Tumor-specific Tc lines were generated by weekly *in vitro* restimulations of $1 \times 10^5$ spleen cells or peritoneal exudate cells from naive or ADJ-PC-5 preimmunized BALB/c mice with $5 \times 10^6$ X-irradiated BALB/c spleen cells and $2 \times 10^5$ X-irradiated ADJ-PC-5 cells in 10 ml MLC medium. After the second to fifth restimulation, most Tc cultures were supplemented with 5% of an IL-2/IFN-$\gamma$ containing supernatant from Con-A-stimulated rat spleen cells (CM) or with 5 U/ml recombinant human IL-2.

### Mixed Lymphocyte Tumor Cell Culture (MLTC)

ADJ-PC-5-specific Tc cells were generated in a primary syngeneic MLTC from spleen cells of naive mice as previously described (Haubeck and Kölsch, 1982). Briefly, $2 \times 10^7$ BALB/c spleen cells were cultured with $1 \times 10^6$ 60-Gy X-irradiated ADJ-PC-5 cells in 10 ml of MLC medium for 6 days. Thereafter, cells were harvested and tested in a 6-hr $^{51}$Cr-release assay for specific cytotoxicity (Haubeck and Kölsch, 1982). In some experiments, the medium used for Tc induction was supplemented with recombinant IL-4, IL-12, or IFN-$\gamma$ or monoclonal antibodies against IL-10.

### *In Vitro* Assay for T-Cell-Mediated Suppression

To test their suppressive capacity, nonadherent PEC from naive and tumor-treated mice, or 30-Gy X-irradiated ADJ-PC-5-specific Tc cells were added to an ADJ-PC-5-specific MLTC (as described earlier) at day 0 of culture. After 6 days, specific cytotoxic activity against ADJ-PC-5 cells was measured in a 6-hr $^{51}$Cr-release assay and compared with the cytotoxicity from cultures without added T cells. For some experiments, IFN-$\gamma$-specific monoclonal antibodies were added to the cultures at various concentrations.

### Flow Cytometric Analysis

For flow cytometric analysis of surface markers on tumor-specific T cells, cells were harvested and washed with BSS. For two-parameter analysis of V$\beta$ expression on CD8$^+$ ADJ-PC-5-specific Tc lines, cells were incubated with FITC-coupled, V$\beta$-specific, and PE-coupled CD8-specific monoclonal antibodies (Pharmingen, San Diego). All washing and incubation steps were performed with ice-cold MLC medium containing 0.1% $NaN_3$. Labeled cells were analyzed using a FACScan flow cytometer (Becton-Dickinson, Heidelberg). Prior to flow cytometric analysis, samples were incubated with 10 $\mu$g/ml propidiumiodide to discriminate dead cells from living cells.

### Sources and Measurement of Cytokines

Recombinant mouse IL-4 and recombinant rat IFN-$\gamma$ were purchased from Hycult Biotechnology (Uden, The Netherlands), whereas recombinant mouse IL-12 was kindly provided by Frank J. Podlasky (Hoffmann-LaRoche, Nutley, NJ). IL-4, IFN-$\gamma$, and IL-10 were determined by means of a sandwich ELISA using 2A5.7 (Sander et al., 1993), and biotinylated XT1 monoclonal anti-bodies for IL-10, R4-6A2 (Spitalny and Havell, 1984), and biotinylated XMG 1.2 for IFN-$\gamma$, 11B11 (Ohara and Paul, 1985), and biotinylated BVD6-24G2 for IL-4. All biotinylated monoclonal antibodies were purchased from Pharmingen (San Diego).

### Detection of Intracellular Cytokines

Staining of intracellular cytokines was performed according to the saponin/monensin method, as described elsewhere (Jung et al., 1993). Briefly, spleen cells, peritoneal exudate cells, or cells from primary MLTC or MLC cultures were purified by centrifugation over a discontinuous Ficoll gradient (Pharmacia, Uppsala, Sweden). Purified cells were stimulated with 10 ng/ml PMA (Serva, Heidelberg) and 1 $\mu$M ionomycin (Sigma, Deisenhofen, Germany) in the presence of 1 $\mu$M monensin (Sigma) for 4 hr. Subsequently, cells were fixed with 4% paraformaldehyde in BSS for 15 min on ice. Intracellular cytokines were stained with biotinylated mAb against murine IFN-$\gamma$ or murine IL-4 (both from Pharmingen) followed by Streptavidin-PE. All incubation steps were performed for 30 min at 4°C. The medium used for incubation and washing steps was BSS containing 5% FCS and 0.1% saponin (Fluka, Neu-Ulm, Germany) to cause reversible pore formation. Immediately before flow cytometric analysis, cells were extensively washed with BSS without saponin to reverse pore formation and counterstained for 30 min with CyChrome-coupled mAb against CD8 and FITC-coupled mAb against V$\beta$6 (both from Pharmingen). Cytokine expression was evaluated with a FACScan flow cytometer (Becton-Dickinson).

### Acknowledgments

This work was supported by the Wilhelm Sander-Stiftung through grant 91.024 and the DFG through SFB 310. C.B. was recipient of a fellowship from the Konrad-Adenauer-Stiftung.

### References

Acha-Orbea H., Zinkernagel R. and Hengartner H. (1985). Cytotoxic T cell clone-specific monoclonal antibodies used to select clonotypic antigen-specific cytotoxic T cells. Eur. J. Immunol. **15**:31-36.

Becker C., Kölsch E. and Pauels H.G. (1997). $CD8^+$ tumor-specific Tc cells primed *in vivo* or *in vitro* against the BALB/c plasmacytoma ADJ-PC-5 use the same TcR $V\beta$ families but display distinct Tc1 or Tc2 characteristics. Immunobiol. **197**:16-30.

Bejarano M.T., de Waal-Malefyt R., Abrams J.S., Bigler M., Bacchetta R., de Vries J.E. and Roncardo M.G. (1992). Interleukin 10 inhibits allogeneic proliferative and cytotoxic T cell responses generated in primary lymphocyte cultures. Int. Immunol. **4**:1389-1397.

Blatt C. and Haïmovich J. (1977). Cell surface immunoglobulins of murine plasma cell tumors. Immunochemistry **14**:687-692.

Bosslet K., Schirrmacher V. and Shantz G. (1979). Tumor metastases and cell-mediated immunity in a model system in DBA/2 mice. VI. Similar specificity patterns of protective anti-tumor immunity *in vivo* and of cytolytic T cells in vitro. Int. J. Cancer **24**:303-313.

Bost K.L., Bieligk S.C. and Jaffe B.M. (1995). Lymphokine mRNA expression by transplantable murine B lymphocytic malignancies. Tumor-derived IL-10 as a possible mechanism for modulating the anti-tumor response. J. Immunol. **154**:718-729.

Browning M.J. and Bodmer W.F. (1992). MHC antigens and cancer: Implications for T-cell surveillance. Curr. Opin. Immunol. **4**:613-618.

Chang W. and Zlotnik A. (1991). IL-10: A novelcytotoxic T cell differentiation factor. J. Immunol. **147**:528-534.

Chen L., Ashe S., Brady W.A., Hellström K.E., Ledbetter J.A., McGowan P. and Linsley P.S. (1992). Costimulation of antitumor immunity by the B7 counterreceptor for the T lymphocyte molecules CD28 and CTL-A4. Cell **71**:1093-1102.

Cihak J., Ziegler H.W. and Kölsch E. (1981). Regulation of immune responses against the syngeneic ADJ-PC-5 plasmacytoma in BALB/c mice. I. Analysis of immune parameters involved. Immunology **43**:133-143.

Fiorentino D.F., Bond M.W. and Mosman T.R. (1989). Two types of mouse T helper cell. IV. Th2 clones secrete a factor that inhibits cytokine production by Th1 clones. J. Exp. Med. **170**:2081-2095.

Gajewski T.F. and Fitch F.W. (1988). Anti-proliferative effect of IFN-$\gamma$ in immune regulation. I. IFN-$\gamma$ inhibits the proliferation of Th2 but not Th1 murine helper T lymphocyte clones. J. Immunol. **140**:4245-4252.

Haskins K., Hannum C., White J., Roehm N., Kubo R., Kappler J. and Marrack F. (1984). The antigen-specific, major histocompatibility complex-restricted receptor on T cells. VI. An antibody to a receptor allotype. J. Exp. Med. **160**:452-471.

Haubeck H.-D. and Kölsch E. (1982). Regulation of immune responses against the syngeneic ADJ-PC-5 plasmacytoma in BALB/c mice. III. Induction of specific T suppressor cells to the BALB/c plasmacytoma ADJ-PC-5 during early stages of tumorigenesis. Immunology **47**:503-510.

Haubeck H.-D. and Kölsch E. (1986). Regulation of immune responses against the syngeneic ADJ-PC-5 plasmacytoma in BALB/c mice. IV. Tumour-specific T suppressor cells, induced at early stages of tumorigenesis, act on the induction phase of the tumour-specific cytotoxic T cell response. Immunobiology **171**:357-365.

Jung T., Schauer U., Heusser C., Neumann C. and Rieger C. (1993). Detection of intracellular cytokines by flow cytometry. J. Immunol. Meth. **159**:197-207.

Kim K.J., Kanellopoulos-Langevin C., Merwin R.M., Sachs D.H. and Asofsky R. (1979). Establishment and characterization of BALB/c lymphoma lines with B cell properties. J. Immunol. **122**:549-554.

LeGros G. and Erard F. (1994). Non-cytotoxic, IL-4, IL-5, IL-10 producing $CD8^+$ T cells: Their activation and effector functions. Curr. Oppin. Immunol. **6**:453-457.

Mengersen R., Schick R. and Kölsch E. (1975). Correlation of "sneaking through" of tumor cells with specific immunological impairment of the host. Eur. J. Immunol. **5**:532-539.

Mosmann T.R., Schumacher J.H., Fiorentino D.F., Leverah J., Moore K.W. and Bond M.W. (1990). Isolation of monoclonal antibodies specific for IL-4, IL-5, IL-6 and a new Th2-specific cytokine synthesis inhibitory factor, by using a solid phase radioimmunoadsorbent assay. J. Immunol. **145**:2938-2945.

Naor D. (1979). Suppressor cells: Permitters and promoters of malignancy. Adv. Cancer Res. **29**:45-125.

North R.J. (1985). Down-regulation of the antitumor immune response. Adv. Cancer Res. **45**:1-43.

Ohara J. and Paul W.E. (1985). Production of a monocloal antibody to and molecular characterization of B cell stimulatory factor-1. Nature **315**:333-336.

Old L.J., Boyse E.A., Clarke D.A. and Carswell E.A. (1962). Antigenic properties of chemically induced tumors. II. Antigens of tumor cells. Ann. N. Y. Acad. Sci. **101**:80-106.

Pauels H.-G., Specht C., Becker C. and Kölsch E. (1996). Suppression of tumor-specific T cell responses against the syngeneic BALB/c plasmacytoma ADJ-PC-5 by tumor induced regulatory T cells via IFN-$\gamma$. Scand. J. Immunol. **43**:421-430.

Ruzek M.C. and Mathur A. (1995). Specific decrease of Th1-like activity in mice with plasma cell tumors. Int. Immunol. **7**:1029-1035.

Sad S., Marcotte R. and Mosman T.R. (1995). Cytokine-induced differentiation of precursor mouse $CD8^+$ T cells into cytotoxic $CD8^+$ T cells secreting Th1 or Th2 cytokines. Immunity **2**:271-279.

Sander B., Höiden I., Andersson U., Möller E. and Abrams J.S. (1993). Similar frequencies and kinetics of cytokine producing cells in murine peripheral blood and spleen: Cytokine detection by immunoassay and intracellular immunostaining. J. Immunol. Meth. **166**:201-214.

Spitalny G.L. and Havell E.A. (1984). Monoclonal antibody to murine gamma interferon inhibits lymphokine-induced antiviral and macrophage tumoricidal activities. J. Exp. Med. **159**:1560-1565.

Sulizeanu D. (1993). Immunosuppressive factors in human cancer. Adv. Cancer Res. **60**:247-267.

